# Outcome of gastric electrical stimulator with and without pyloromyotomy for refractory gastroparesis

**DOI:** 10.1007/s00464-024-11099-w

**Published:** 2024-08-07

**Authors:** Pauline Aeschbacher, Angelica Garcia, Justin Dourado, Peter Rogers, Garoufalia Zoe, Ana Pena, Samuel Szomstein, Emanuele Lo Menzo, Raul Rosenthal

**Affiliations:** 1 Department of General Surgery and the Bariatric and Metabolic Institute, Cleveland Clinic Florida, 2950 Cleveland Clinic Blvd, Weston, FL 33331 USA; 2 Department of Visceral Surgery and Medicine, Inselspital, Bern University Hospital, University of Bern, Bern, Switzerland; 3 Ellen Leifer Shulman and Steven Shulman Digestive Diseases Centre, Cleveland Clinic Florida, 2950 Cleveland Clinic Blvd., Weston, FL 33331 USA

**Keywords:** Gastric electrical stimulator, Pyloromyotomy, Gastroparesis, Refractory gastroparesis, Surgical treatment, Treatment

## Abstract

**Background:**

Surgical treatments of refractory gastroparesis include pyloromyotomy and gastric electrical stimulator (GES). It is unclear if patients may benefit from a combined approach with concomitant GES and pyloromyotomy.

**Methods:**

Retrospective cohort analysis of all patients with refractory gastroparesis treated with GES implantation with and without concomitant pyloromyotomy at Cleveland Clinic Florida from January 2003 to January 2023. Primary endpoint was efficacy (clinical response duration and success rate) and secondary endpoints included safety (postoperative morbidity) and length of stay. Success rate was defined as the absence of one of the following reinterventions during follow-up: Roux-en-Y gastric bypass (RYGB), pyloromyotomy, GES removal.

**Results:**

During a period of 20 years, 134 patients were treated with GES implantation. Three patients with history of previous surgical pyloromyotomy or RYGB were excluded from the analysis. Median follow-up was 31 months (IQR 10, 72). Forty patients (30.5%) had GES with pyloromyotomy, whereas 91 (69.5%) did not have pyloromyotomy. Most of the patients had idiopathic (*n* = 68, 51.9%) or diabetic (*n* = 58, 43.3%) gastroparesis. Except for preoperative use of opioids (47.5 vs 14.3%; *p* < 0.001), patient’s characteristics were similar in both groups. There were no significant differences between the two groups in terms of overall postoperative complications (17.5% vs 14.3%; *p* = 0.610), major postoperative complications (0% vs 2.2%; *p* = 1), and length of stay (2(IQR 1, 2) vs 2(IQR 1, 3) days; *p* = 0.068). At 5 years, success rate was higher in patients with than without pyloromyotomy however not statistically significant (82% versus 62%, *p* = 0.066). Especially patients with diabetic gastroparesis seemed to benefit from pyloromyotomy during GES (100% versus 67%, *p* = 0.053). In an adjusted Cox regression, GES implantation without pyloromyotomy was associated with a 2.66 times higher risk of treatment failure compared to GES implantation with pyloromyotomy (HR 2.66, 95% CI 1.03–6.94, *p* = 0.044).

**Conclusion:**

Pyloromyotomy during GES implantation for gastroparesis seems to be associated with a longer clinical response with similar postoperative morbidity and length of hospital stay than GES without pyloromyotomy. Patient with diabetic gastroparesis might benefit from a combination of GES implantation and pyloromyotomy.

Gastroparesis is a rare disease consisting of delayed gastric emptying in the absence of mechanical obstruction [1, 2]. The prevalence of gastroparesis is estimated between 13.8 and 24.2 per 100,000 persons in Western countries [3]. However, the methods used to diagnose gastroparesis, such as gastric emptying studies, are rarely performed if gastroparesis is not suspected, and the prevalence might be higher. Symptoms usually include upper abdominal pain, early satiety, nausea, vomiting, belching, or bloating. Diabetes mellitus is a common cause of gastroparesis, but a large proportion of cases are idiopathic [4].

There is currently no consensus on the underlying physiopathology of gastroparesis [1, 2]. It might be associated with dysfunction in the gastric pacemaker cells known as interstitial cells of Cajal (ICC), impairment in slow wave propagation, and their interaction with other components involved in regulating gastric motor function [5].

The management of gastroparesis is primarily conservative with dietary modification, nutritional support, medication (prokinetics, anti-emetic, neuromodulators, proton pump inhibitor, analgesics) and optimal glucose control for diabetic gastroparesis [1, 4, 6]. Despite the availability of various treatment modalities, a subset of patients with refractory gastroparesis continues to suffer from debilitating symptoms and impaired quality of life [1]. Around 20–30% of patients have no symptom improvement after medical treatment and nutritional support and are considered to have medically refractory gastroparesis [4]. In the case of refractory gastroparesis, a surgical approach should be evaluated.

Over the past two decades, gastric electrical stimulation (GES) has emerged as a promising therapeutic option for these patients [4, 7]. The current GES device used is the Enterra® Therapy (Medtronic Corp., Minneapolis, MN), which was FDA-approved based on humanitarian device exemption in 2000. Since then, it has been proven effective in treating gastroparesis-related symptoms, especially in reducing vomiting and nausea [8]. It is, however, less effective in bloating sensations and abdominal pain. Success rates vary from 45 to 90%, depending on the study [4, 9–12]. A concomitant pylorotomy has been proposed to improve the success rate [13]. However, its superiority over GES or pyloromyotomy alone is controversial due to the scarcity of data on the subject and poor long-term follow-up [13–15]. Furthermore, the morbidity linked to the association of both interventions during one surgery is unknown.

This study aims to evaluate the clinical response duration and perioperative outcomes of GES implantation with pyloromyotomy as a possible surgical alternative to GES implantation alone.

## Methods

### Patients’ selection and data analysis

Following approval by our Institutional Review Board (FLA 23-010), all patients with refractory gastroparesis treated with GES implantation between 01/01/2003 and 01/01/2023 were retrospectively reviewed. Patients with a history of previous surgical pyloromyotomy, G-POEM, RYGB, or subtotal gastrectomy prior to GES implantation were excluded. A medical record review of all patients meeting the inclusion criteria was performed, and patients’ characteristics, surgical outcomes, and follow-up were reported. Patients were divided according to the presence or absence of concomitant surgical pyloromyotomy during GES implantation. The STROBE checklist was used to report our methodology and findings.

### Indication

The first-line treatment for patients with proven gastroparesis encountered at our clinic typically involves dietary modification, nutritional support if necessary, and medication. Patients presenting with refractory gastroparesis are usually evaluated for GES implantation or pyloromyotomy. If the benefit of GES implantation is unclear, temporary external GES placement might be discussed with the patient.

### Surgical technique

At our clinic, GES implantations (Enterra Therapy, Medtronic Corp, Milwaukee, WI) are performed laparoscopically by a single surgeon (senior author). Under endoscopic surveillance 2 electrodes are inserted into the seromuscular layer of the greater curvature of the stomach, 20 cm proximal of the pylorus and at least 2 cm apart from each other. After fixing the electrodes to the gastric wall, they are externalized and connected to the battery located in a prepared subcutaneous pocket in the left lower abdomen and fixed to the muscle fascia. If a pyloromyotomy is planned, it is performed prior to GES implantation as described by Heineke-Mikulicz [16]. A longitudinal incision with division of the longitudinal and circular muscle layers through the pylorus is made with an extension from the antrum to the duodenum pars I. Gentle traction on the edges of the incision is applied before performing a transverse closure with resorbable sutures.

### Study endpoints

The primary endpoint was efficacy with duration of clinical response and success rate. The secondary outcomes were postoperative morbidity and length of stay. Success rate was defined as the absence of one of the following reinterventions during follow-up: RYGB, pyloromyotomy, GES removal.

### Statistical analysis

Descriptive statistics were used. Categorical variables were presented as numbers and percentages; continuous variables were expressed as median and interquartile range or mean and standard deviation as appropriate. Comparisons between the groups (GES with or without pyloromyotomy) were performed using the Fisher exact test and Chi-square test for categorical variables and Mann–Whitney-*U* test and Student-*t*-test for continuous variables, as appropriate. Success interval was defined as the time from GES implantation to the date of the first reintervention for RYGB, pyloromyotomy, or GES removal or the last follow-up in months. Survival analyses were conducted using Kaplan–Meier statistics and Log-rank test. Cox regression analysis adjusted for known risk factors associated with poorer prognosis of gastroparesis (including idiopathic and postsurgical etiology, obesity, chronic abdominal pain, and opioid use) was performed [2]. Statistical analyses were done using EZR (version 1.61) and R software (version 4.3.1).

## Results

### Patient selection

From January 1, 2003, to January 1, 2023, 134 patients underwent GES implantation for refractory gastroparesis. Three patients with a history of previous surgical pyloromyotomy or RYGB were excluded from the analysis, resulting in a total of 131 included patients. Of these, 69.5% (*n* = 91) underwent GES implantation alone, and 30.5% (*n* = 40) underwent concomitant pyloromyotomy. The median follow-up was 31 months (IQR 10, 72). Follow-up was available for 79% (*n* = 104) patients at 12 months (78% with and 80% without pyloromyotomy), 65% (*n* = 85) at 24 months (68% with and 64% without pyloromyotomy) and 55% (*n* = 72) at 36 months (60% with and 53% without pyloromyotomy).

### Patient characteristics

The cohort had a median age of 46 (IQR 32, 54) and a median body mass index (BMI) of 23.9 kg/m^2^ (IQR 20.4, 28). Twenty-nine patients were male (22.1%). All patient characteristics are summarized in Table 1. Except for a higher frequency of preoperative opioids in the GES implantation with pyloromyotomy group (47.5% vs 14.3%, *p* < 0.001), no differences in patient characteristics were observed.

**Table 1:** Patients characteristics according to the type of surgery (gastric electrical stimulator implantation with versus without pyloromyotomy)

	GES with PP	GES w/o PP	*p*-value
	*n* = 40	*n* = 91	
Sex			
Female	30 (76.9)	71 (78.0)	1
Male	9 (23.1)	20 (22.0)	
Race			
White	28 (71.8)	54 (62.8)	0.634
Hispanic	7 (17.9)	12 (14.0)	
African American	3 (7.7)	15 (17.4)	
Native American	0 (0.0)	1 (1.2)	
Asian	0 (0.0)	2 (2.3)	
Other	1 (2.6)	2 (2.3)	
Age	47 (11.2)	42 (15.7)	0.093
BMI	22.9 (20.9–28.6)	24.3 (19.9–27.4)	0.913
BMI > 30 kg/m^2^	8 (20.5)	13 (17.6)	0.800
Smoking			0.222
Active	3 (7.5)	12 (13.2)	
Stopped	8 (20.0)	11 (12.1)	
Never	28 (70.0)	58 (63.7)	
Unknown	1 (2.5)	10 (11.0)	
Etiology			0.182
Idiopathic	26 (65.0)	42 (46.2)	
Diabetic	14 (35.0)	44 (48.4)	
Postsurgical	0 (0.0)	2 (2.2)	
Other	0 (0.0)	3 (3.3)	
Preoperative medication			
Antacid	33 (82.5)	61 (67.0)	0.092
Antiemetic	31 (77.5)	57 (62.6)	0.109
Opioids	19 (47.5)	13 (14.3)	** < 0.001**
Comorbidity			
Diabetes mellitus	14 (35.0)	46 (50.2)	0.128
Hypertension	11 (27.5)	31 (34.1)	0.544
Cardiac disease	8 (20.0)	12 (13.2)	0.429
Respiratory disease	8 (20.0)	13 (14.3)	0.443
Neurological disease	8 (20.0)	12 (13.2)	0.429
Psychological disease	6 (15.0)	18 (19.8)	0.628
Renal disease	5 (12.5)	10 (11.0)	0.773
Chronic pain	5 (12.5)	4 (4.4)	0.131
Liver disease	1 (2.5)	1 (1.1)	0.519
Previous history			
Pyloric botox injection	7 (17.5)	12 (13.2)	0.592
Pyloric dilatation	0 (0.0)	1 (1.1)	1
Hiatal hernia repair	1 (2.5)	2 (2.2)	1
Feeding jejunostomy	6 (15.0)	6 (6.6)	0.185
Venting gastrostomy	1 (2.5)	6 (6.6)	0.675

*n* (%) for categorical variable and median (interquartile range) or mean (standard deviation) for continuous variable, statistically significant *p* value (*p* < 0.05) are marked in bold.

*BMI* body mass index, *GES* gastric electrical stimulator, *PP* pyloromyotomy

### Success rate

**Fig. 1:**
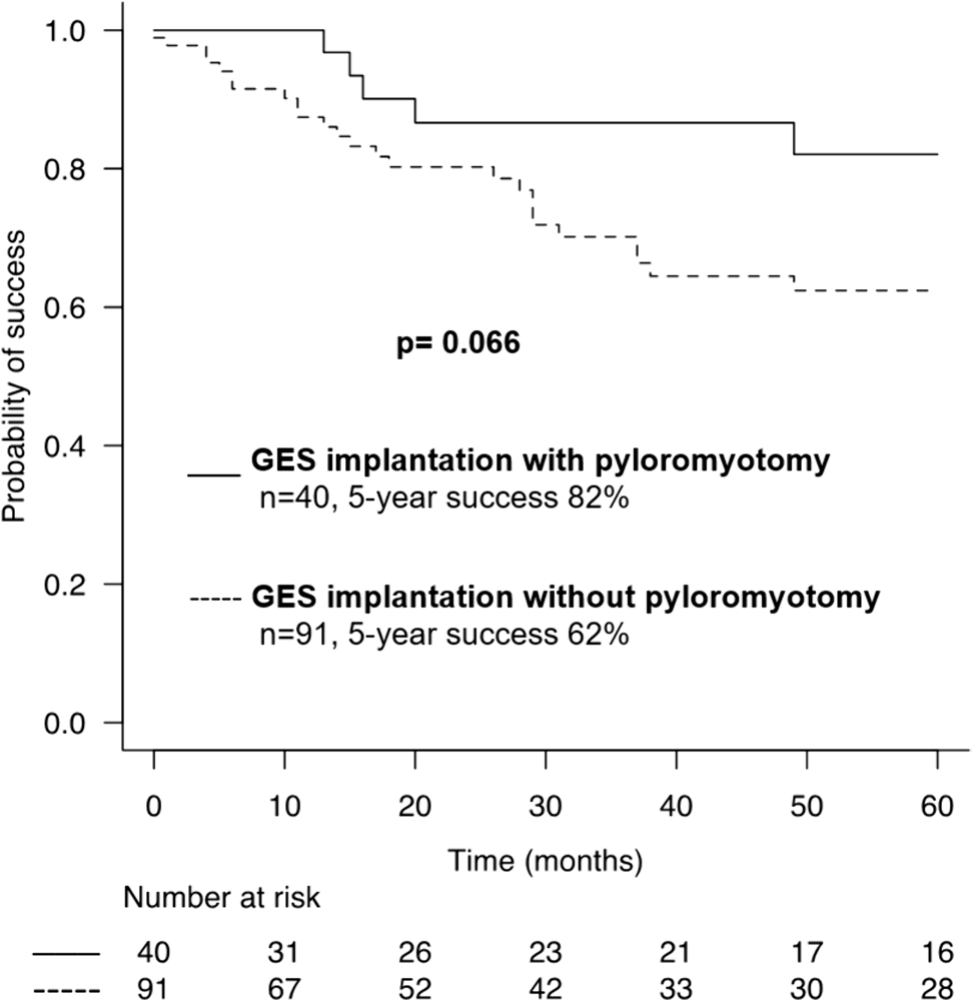
Success rate (%) at follow-up (months) after gastric electrical stimulator implantation with or without pyloromyotomy

Kaplan–Meier curve analysis showed a trend for a higher clinical response rate at 5 years after GES with pyloromyotomy (82%) versus without (62%), without statistical significance (*p* = 0.066) (Fig. 1). At follow-up, 2.5% (*n* = 1) of patients with GES with pyloromyotomy and 6.6% (*n* = 6) of those with GES without pyloromyotomy were converted to RYGB (*p* = 0.675). Fifteen patients (16.5%) underwent pyloromyotomy or G-POEM at follow-up after GES without pyloromyotomy. There were 25 GES explantations at follow-up, 5 after GES with pyloromyotomy and 20 after GES without pyloromyotomy (*p* = 0.236).

**Fig. 2:**
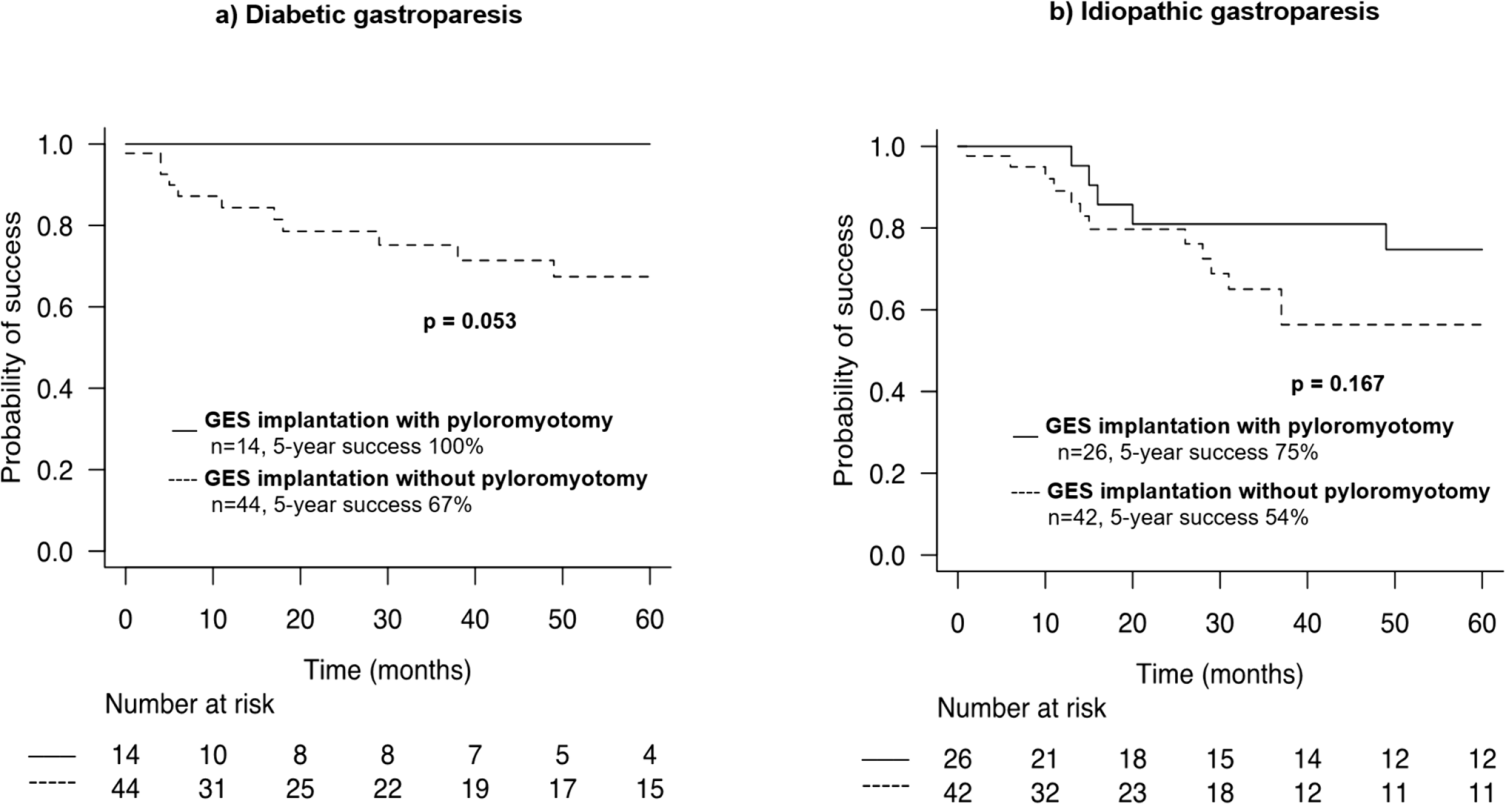
Success rate (%) at follow-up (months) after gastric electrical stimulator implantation with or without pyloromyotomy (**a**) for patient with diabetic gastroparesis (**b**) for patient with idiopathic gastroparesis

Looking at the clinical response according to gastroparesis etiology, patients with diabetic gastroparesis seemed to benefit more from GES with pyloromyotomy (5-year success rate 100% versus 67%, *p* = 0.053) than patients with idiopathic gastroparesis (5-year success rate 75% vs 54%, *p* = 0.167) (Fig. 2a, b).

In a Cox regression analysis adjusted for known risk factors associated with treatment failure (idiopathic and postsurgical etiology, obesity, chronic abdominal pain, and opioid use), GES implantation without pyloromyotomy was associated with a 2.66 times higher risk of treatment failure compared to GES implantation with pyloromyotomy (HR 2.66, 95% CI 1.03–6.94, *p* = 0.044).

### Perioperative outcomes

When comparing GES implantation with or without pyloromyotomy, no differences were observed in terms of length of stay, readmission, overall and major postoperative complications (Table 2). No leakage was observed after pyloromyotomy and surgical site infection occurred in one patients of each group requiring no GES explantation (2.5% vs 1.1%, *p* = 0.519). Two patients required relaparoscopy at 30 days in the GES implantation without pyloromyotomy group, but this difference was not statistically significant (0% vs 2.2%, *p* = 1). No other reinterventions or reoperations were observed at 30 days postoperatively.

**Table 2:** Postoperative outcomes according to the type of surgery (gastric electrical stimulator implantation with versus without pyloromyotomy)

	GES with PP	GES w/o PP	*p*-value
	*n* = 40	*n* = 91	
Length of stay	2 (1–2)	2 (1–3)	0.068
Readmission	6 (15.0)	5 (5.5)	0.09
Overall postoperative complication	7 (17.5)	13 (14.3)	0.610
Major postoperative complication	0 (0.0)	2 (2.2)	1
Abdominal pain	2 (5.0)	4 (4.4)	1
SSI (organ/space)	1 (2.5)	1 (1.1)	0.519
Bleeding/hematoma	0 (0.0)	2 (2.2)	1
Pneumonia	0 (0.0)	1 (1.1)	1
Thrombosis/Lung emboli	1 (2.5)	0 (0.0)	0.303
Other complication	4 (10.0)	5 (5.5)	0.454
Reoperation	0 (0.0)	2 (2.2)	1
Postoperative mortality	0 (0.0)	1 (1.1)	1
Follow-up (months)	46 (14;73)	44 (15;99)	0.358
Success rate	34 (85.0)	59 (64.8)	**0.022**
Roux-en-Y gastric bypass at follow-up	1 (2.5)	6 (6.6)	0.675
Pyloromyotomy during follow-up	0 (0.0)	13 (14.3)	**0.01**
G-POEM	0 (0.0)	2 (2.2)	1
Laparoscopic pyloromyotomy	0 (0.0)	11 (12.1)	0.018
GES explantation during follow-up	5 (12.5)	20 (22.0)	0.236
Pain	4 (10.0)	10 (11.0)	1
Inefficacy	3 (7.5)	7 (7.7)	1
Infection	0 (0.0)	2 (2.2)	1
Other	0 (0.0)	4 (4.4)	0.313

*n* (%) for categorical variable and median (interquartile range) or mean (standard deviation) for continuous variable, statistically significant *p* value (*p* < 0.05) are marked in bold.

*GES* gastric electrical stimulator, *G-POEM* gastric peroral endoscopic myotomy, *PP* pyloromyotomy, *SSI* surgical site infection

## Discussion

Our current retrospective single-center comparison of GES implantation with versus without pyloromyotomy highlighted similar postoperative outcomes with a trend for a longer clinical response with concomitant pyloromyotomy, especially for diabetic patients.

Gastroparesis can cause significant patient discomfort, leading to persistent nausea and vomiting, weight loss, bloating, and early satiety [4]. Although multiple treatment options have been proposed for gastroparesis, many patients remain unresponsive to medical therapy and require additional interventions to alleviate symptoms. Current treatment options for refractory gastroparesis consist of gastric stimulator implantation (GES), surgical or per-oral endoscopic pyloromyotomy (PP, POP, or G-POEM) [2, 6, 17]. Total and subtotal gastrectomy have also been reported in the literature. However, due to the invasive nature of such a procedure, it is more of a last-resort option [6]. A venting gastrostomy might be considered to decompress the stomach for symptom improvement [6]. Other less common and less effective treatment options include intrapyloric botulinum toxin injection, transpyloric stenting, or pyloric dilatation. There is currently no consensus on which approach is the most appropriate and effective for gastroparesis treatment [1, 6]. A discrepancy is often observed between patients’ reported symptoms and satisfaction and findings in functional diagnostics such as gastric emptying [8, 17, 18].

Gastric electrical stimulation consists of pacing the stomach with the propagation of slow waves from the greater curvature toward the pylorus [4]. Although the exact physiological mechanism of action of the gastric stimulator is not fully understood, studies have shown that high-frequency stimulation can enhance the amplitude and propagation velocity of the slow waves while reducing nausea and vomiting, probably through the activation of vagal afferent pathways and by increasing the maximum tolerated gastric volume [12, 17]. With this, gastric electrical stimulation (GES) has emerged as an effective treatment for patients with refractory gastroparesis, especially those with diabetes. However, concerns about its effectiveness have been raised, as the one-year clinical response rate ranges from 45 to 90% [3]. Pyloromyotomy is also an effective treatment for refractory gastroparesis and has been reported feasible as a first-line intervention or after GES implantation [19, 20]. A handful of small series also reports the outcome of GES implantation with concomitant pyloromyotomy. Davis et al. reported accelerated gastric emptying and symptoms reduction after GES implantation with pyloromyotomy in a single cohort of 24 patients [21]. Sarosiek et al. highlighted similar results, this time when compared to GES alone [13]. Zoll et al. reported higher nausea and vomiting improvement in 21 GES implantation with pyloromyotomy when compared to 74 GES and 25 pyloric interventions [14]. On the other hand, Marowski et al. highlighted no benefit from a combination of both procedures [15]. Concerns might be raised about the comorbidity of performing a pyloromyotomy while increasing gastric motility and the risk of leakage or device infection that it might ensure. To add data on the safety of performing GES with pyloromyotomy, our study demonstrates similar postoperative morbidity and length of stay, and readmission when compared to GES alone. Regarding the need for a reoperation, we highlighted a trend toward lower reoperation after GES with pyloromyotomy; however, this result was not significant. Given the chronic and progressive nature of the condition, adopting a stepwise approach could be an alternative, particularly in younger patients. However, it remains to be determined which patients would benefit more from a combined procedure versus a stepwise approach.

Previous studies reported better GES efficacy in diabetic gastroparesis than in idiopathic gastroparesis [5, 22]. A review published by Chu et al. involving 601 subjects concluded that the beneficial effects of GES were seen more in patients with diabetic gastroparesis than in those with post-surgical and idiopathic etiologies [2, 23]. Interestingly, the etiology of gastroparesis does not seem to have an impact on the outcomes after pyloromyotomy [24]. Looking at our results, a combination of GES and PP seems to be more effective for the specific cohort of diabetic gastroparesis.

The rate of GES explantation observed in this study was 19%, with device-related pain being the most common reason for explantation. The inefficacy of the device was another significant reason for explantation, suggesting that GES may not be universally effective in all patients with refractory gastroparesis or may have a limited efficacy duration. Although not significant, our cohort highlighted a trend toward fewer GES explantations after GES implantation with pyloromyotomy. These results need to be confirmed by additional data.

Our study presents certain limitations due to its small sample size which might lead to a type II errors (false negatives). Further studies with larger sample size are needed to evaluate the potential benefit of a concomitant pyloromyotomy during GES implantation. Although both groups are similar, there is a higher rate of opioid use in GES implantation with pyloromyotomy, which is associated with worse outcomes [2]. The cox regression analysis was adjusted for this difference. Our study did not report patients’ related outcomes, medication changes and data on functional diagnostics (gastric emptying) at follow-up. The treatment success was based on the absence of reoperation for gastroparesis and is limited by the follow-up duration. Therefore, these results should be taken with caution until stronger data on the combination of GES implantation and pyloromyotomy are available. Nevertheless, GES implantation with pyloromyotomy seems safe and feasible and might bring additional benefits in the long-term outcomes of patients.

## Conclusion

GES implantation with pyloromyotomy for refractory gastroparesis is safe, with similar postoperative morbidity and length of hospital stay compared to GES implantation alone. Clinical response seems to be longer in patient with GES implantation and pyloromyotomy. Patients with diabetic gastroparesis may particularly benefit from the combined approach of GES implantation and pyloromyotomy.
